# Meningococcal Serogroup A, C, W_135_ and Y Conjugated Vaccine: A Cost-Effectiveness Analysis in the Netherlands

**DOI:** 10.1371/journal.pone.0065036

**Published:** 2013-05-31

**Authors:** Hiltsje Hepkema, Koen B. Pouwels, Arie van der Ende, Tjalke A. Westra, Maarten J. Postma

**Affiliations:** 1 Unit of PharmacoEpidemiology and PharmacoEconomics (PE2), Department of Pharmacy, University of Groningen, Groningen, The Netherlands; 2 Academic Medical Center, Center for Infection and Immunity Amsterdam (CINIMA), Department of Medical Microbiology and the Netherlands Reference Laboratory for Bacterial Meningitis, Amsterdam, The Netherlands; 3 GlaxoSmithKline, Corporate Affairs, Zeist, The Netherlands; Health Protection Agency, United Kingdom

## Abstract

**Background:**

In 2002, vaccination with a serogroup C meningococcal conjugate vaccine (MenC) was introduced in the Netherlands for all children aged 14 months. Despite its success, herd immunity may wane over time. Recently, a serogroup A,C,W135,Y meningococcal conjugate vaccine (MenACWY) was licensed for use in subjects of 12 months of age and above.

**Objectives:**

To evaluate the cost-effectiveness of meningococcal vaccination at 14 months and an additional vaccination at the age of 12 years, both with the MenACWY vaccine.

**Methods:**

A decision analysis cohort model, with 185,000 Dutch newborns, was used to evaluate the cost-effectiveness of different immunization strategies. For strategies including a vaccination at 12 years of age, an additional cohort with adolescents aged 12 years was followed. The incremental cost-effectiveness ratio (ICER) was estimated for the current disease incidence and for a scenario when herd immunity is lost.

**Results:**

Vaccination with MenACWY at 14 months is cost-saving. Vaccinating with MenACWY at 14 months and at 12 years would prevent 7 additional cases of meningococcal serogroup A,C,W135,Y disease in the birth cohort and adolescent cohort followed for 99 years compared to the current vaccine schedule of a single vaccination with MenC at 14 months. With the current incidence, this strategy resulted in an ICER of €635,334 per quality adjusted life year. When serogroup C disease incidence returns to pre-vaccination levels due to a loss of vaccine-induced herd-immunity, vaccination with MenACWY at 14 months and at 12 years would be cost-saving.

**Conclusions:**

Routine vaccination with MenACWY is cost-saving. With the current epidemiology, a booster-dose with MenACWY is not likely cost-effective. When herd immunity is lost, a booster-dose has the potential of being cost-effective. A dynamic model should be developed for more precise estimation of the cost-effectiveness of the prevention of disappearance of herd immunity.

## Introduction

*Neisseria meningitidis* (the meningococcus) is an important cause of bacterial meningitis and septicaemia worldwide [1], [2]. Meningococcal disease has a high mortality rate and survivors are at high risk of having permanent sequelae like mental retardation, hearing loss, scars and amputations [3], [4].

In the Netherlands, routine vaccination at the age of 14 months with a meningococcal serogroup C conjugate (MenC) vaccine was implemented in the National Immunization Program (NIP) after a meningococcal serogroup C disease outbreak in 2000 and 2001. This implementation was accompanied by a catch-up program for all children and adolescents aged 1 to 18 years. Hereafter, the incidence of serogroup C disease decreased considerably [5] and probably also carriage of serogroup C strains of *Neisseria meningitidis* decreased as seen in the United Kingdom (UK) [6], [7].

The decreased carriage of serogroup C meningococci has led to a reduced transmission of the bacterium whereby unvaccinated individuals are protected. This herd immunity effect might be partly the reason for the decreased incidence of serogroup C disease in the Netherlands [8]. High vaccination coverage in the age group with a likely high meningococcal transmission rate, i.e. adolescents [9], is a key factor for achieving herd protection [2]. Therefore, the catch-up program likely accounts for the majority of the herd immunity effect. However, the adolescent population of the future may have levels of antibodies which are too low to be protective against carriage and/or disease since they are vaccinated at the age of 14 months and duration of protection is limited when vaccinating at young age [10]. An unprotected adolescent population could result in a renewed circulation of serogroup C meningococci and a considerable reduction in herd immunity. Therefore, it might be necessary to add a booster-dose early in adolescence to maintain the herd immunity effect and provide protection against meningococcal disease in this age group [11], [12].

Consequently, Austria and Switzerland have recently added a booster-dose with a conjugated MenACWY or MenC vaccine for children around the age of 12 years in addition to infant immunization [13], [14].

While the incidence of serogroup C meningococcal disease is currently still low in the Netherlands, the incidence of serogroup Y disease is increasing, especially in adolescents (Appendix S1) [15]. The quadrivalent conjugate vaccine against serogroup A, C, W135 and Y disease (MenACWY) indicated for use in subjects of 12 months of age or older (Nimenrix, GlaxoSmithKline) was recently licensed in the European Union [16]. This vaccine is the only quadrivalent vaccine that is currently licensed in the European Union for use in children younger than two years of age and could be used to replace the current MenC vaccination among Dutch children aged 14 months. The shift from a MenC vaccine to this MenACWY vaccine in the NIP might prevent additional cases of meningococcal disease.

In this paper, we present the economic evaluation of routine vaccination with the MenACWY vaccine at 14 months and the evaluation of an additional booster-dose with MenACWY early in adolescence.

## Methods

Health effects, costs, savings and the incremental cost-effectiveness ratios (ICERs) of routine vaccination with the MenACWY vaccine for infants aged 14 months and of an additional booster-dose of the MenACWY vaccine at the age of 12 years were estimated. These strategies are further named ‘MenACWY’ for only vaccinating at 14 months, and ‘MenACWY+MenACWY’ for vaccinating at 14 months and 12 years. Both strategies were compared with the current situation in the Netherlands: vaccinating with MenC at 14 months (‘MenC’). The booster-dose strategy was also compared with MenACWY at 14 months.

According to the Dutch guidelines for pharmacoeconomic research [17], the study was performed from the societal perspective. Future costs and health effects were discounted with respectively 4% and 1.5%. ICERs were calculated by dividing incremental costs in euros (€) by quality adjusted life years (QALYs) gained. All calculations were carried out with Excel 2010 (Microsoft).

### Model

**Figure 1:**
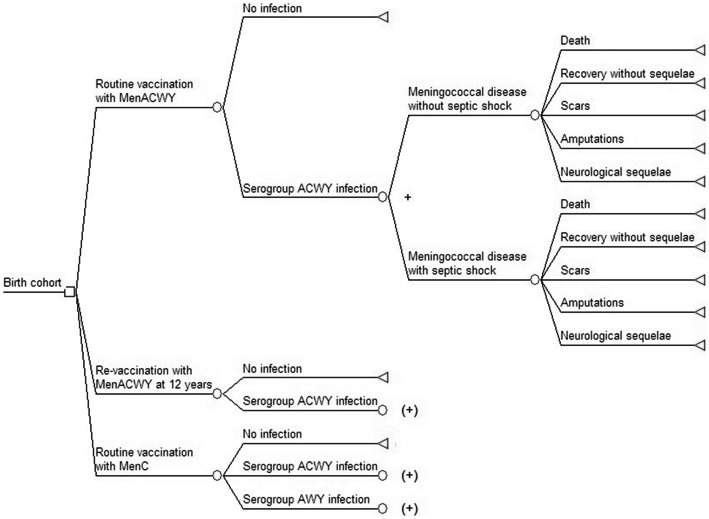
Decision tree used in the economic model. Squares represent decision nodes, circles represent probabilities and triangles represent end states. (+) indicates that the decision tree follows the same path as the branch indicated with +.

A decision tree analytic model was designed to simulate the different vaccination strategies (fig. 1). A cohort of 185,000 Dutch newborns [18], was run through the decision tree once per strategy and for the comparator. For estimation of the ICERs of vaccinating at 12 years of age, an additional cohort of 200,000 12-year old adolescents was followed [18]. For this separate cohort of 12-year old adolescents, it was assumed that protection by vaccination at the age of 14 months was still partly present. In addition, we removed all children who had meningococcal disease before the age of 12 years from this cohort. Time cycles of 1 month for children less than 2 years of age and annual cycles for children above this age were used. The time horizon of the study was 99 years, taking lifetime costs and effects into account. The parameters used in the model are shown in table 1 and table 2.

**Table 1:** Probability parameters used in the economic model.

	Base case^a^	Distribution	References		
**Vaccine parameters**					
*Vaccine efficacy (%)*					
Initial vaccine efficacy (14 months)	89.96	Triangular (64;89.96;98)		[29], [30]	
Initial vaccine efficacy (12 years)	95.95	Triangular (92;95.95;99)		[29], [30]	
*Adverse events*					
Number of AE per 10,000 immunizations	3.48	Beta (4764;13691736)		[48]	
Number of AR per 10,000 immunizations	0.02	Beta (27;13696473)		[48]	
*Vaccination coverage (%)*					
14 months	95.9	Fixed		[49]	
12 years	94.0	Fixed		[50]	
**Disease parameters**					
ACWY incidence (per 100,000 persons)	0.15 (age dependent)	Beta (25;16431651)		[15]	
*Clinical features (%)*					
Meningococcal disease	65.1	Beta (443;238)		[51], [52]	
Meningococcal disease with septic shock	34.9	Dependent of MD		[51], [52]	
*Case fatality (%)*					
Case fatality rate MD	2.1	Beta (8;372)		[51]	
Case fatality rate MDS	13.0	Beta (21;140)		[51]	
*Sequelae (%)*					
Scars	2.8	Beta (16;546)		[4]	
Amputations	0.7	Beta (4;558)		[4]	
Neurological sequelae	6.9	Beta (127;1707)		[3], [4], [39]	
**Demographic parameters**					
Birth cohort	185,000	Fixed		[18]	
Adolescent cohort (aged 12 years)	200,000	Fixed		[18]	
Probability of being a girl	49.0%	Fixed		[18]	
Probability of being a boy	51.0%	Fixed		[18]	
Probability of dying of other causes	Age dependent	Fixed		[15], [18]	

AE = adverse event, AR = Anaphylactic reactions, MD = meningococcal disease without septic shock, MDS = meningococcal disease with septic shock.

a Values are presented as percentages or rates.

**Table 2:** Unit costs for resource utilization in euros (€) and other parameters used in the economic model.

	Base case	Distribution	References
**Cost parameters (€)**			
*Overhead costs of the immunization program*			
Routine immunization of toddlers	62,476	Fixed	[20]
Booster-dose at the age of 12 years	62,476	Fixed	[20]
*Vaccine dose*			
MenACWY	42.72	Fixed	[23]
MenC	55.11	Fixed	[24]
*Other costs*			
Administration costs routine infant vaccination	6.30	Fixed	[20]
Administration costs booster dose	14.50	Fixed	[20]
Booster-dose boys invitation	0.50	Fixed	[20]
GP visit	29	Fixed	[17]
Microbiological diagnostics and MRI	305	Gamma (1;305)	[20], [53]
Full course of parenteral antibiotic treatment	297	Gamma (1;297)	[20]
Inpatient day (general ward)	463	Fixed	[17], [54]
Inpatient day (intensive care unit)	2,263	Fixed	[17]
Extra medical assistance with septic shock	1,879	Gamma (1;1879)	[20]
Treatment for scars	506	Gamma (1;506)	[20]
Treatment for amputations	1,628	Gamma (1;1628)	[20]
Institutional care (annual costs)	90,036	Gamma (1;90036))	[17]
Special education (annual costs)	4,700–13,810	Gamma (age dependent)	[18], [22]
Pediatrician follow-up	122	Gamma (1;122)	[20]
Public health follow-up	62	Gamma (1;62)	[20]
*Productivity costs (per hour)*			
Parents	28.74	Gamma (1;29)	[17], [18]
Children in the cohort	9.51–37.34	Gamma (age dependent)	[17]
**Other parameters**			
*Duration of hospitalization (days)*			
Standard hospitalization MD	15	Gamma (1;15)	[20]
Standard hospitalization MDS	13	Gamma (1;13)	[20]
Intensive care unit MDS	5	Gamma (1;5)	[20]
Treatment of scars	2	Gamma (1;2)	[20]
Treatment of amputations	8	Gamma (1;8)	[20]
*Total drop in quality of life (QALY)*			
Amputations or scars	0.17	Beta (5.20;25.39)	[21]
Neurological sequelae	0.25	Beta (10.01;30.02)	[21]
Average quality of life general population	0.89	Beta (age dependent)	[55]
*Discount rates (%)*			
Costs	4.0	Fixed	[17]
Health effects	1.5	Fixed	[17]

HPV = human papillomavirus, GP = general practitioner, MD = meningococcal disease without septic shock, MDS = meningococcal disease with septic shock, MRI = magnetic resonance imaging scan, QALY = quality adjusted life year, NA = not applicable.

### Epidemiology

The incidence of meningococcal serogroup A,C,W135,Y disease was determined using data from the Netherlands Reference Laboratory for Bacterial Meningitis (NRLBM, Academic Medical Center, Amsterdam) (appendix S1) [15], [19]. For the base-case, the average incidence of 2007–2011 was used. For scenario analyses, the serogroup ACWY incidence of 2011 and the serogroup C incidence of 2001 were used. Incidences of 2007–2011 and of 2001 were adjusted for respectively a reporting percentage of 85–90% and 83.3% [15], [20].

### Healthcare Resource Use

Based on our previous study [20], we assumed that meningococcal disease without a septic shock requires one general practitioner visit, microbiological diagnostics, a magnetic resonance imaging scan, antibiotic treatment, 15 days hospitalization and follow-up visits after recovery. This follow-up includes 2 outpatient consultations of a paediatrician for patients aged less than 15 years and two general practitioner visits for patients 15 years or older [20]. When disease is accompanied by a septic shock, instead of 15 days hospitalization, there is a need for 5 days intensive care unit, extra medical assistance and 13 days hospitalization [20].

Patients with meningococcal disease can recover completely, but the disease can also result in death or sequelae like neurological sequelae, scars or amputations. We assumed that 25% of the patients with neurological sequelae (i.e. hearing loss, mental retardation) require lifetime intensive institutional care and 50% of the patients require special education [20]. Other resource utilization taken into account is the public health follow-up of a meningococcal case [20].

QALY losses due to permanent sequelae, i.e. amputations, scars and neurological sequelae were included in the analyses [21]. We did not take into account QALY losses associated with acute forms of disease or minor vaccine-related adverse events, because of the limited duration of these events.

### Costs

Direct and indirect costs were considered at 2011 price levels (table 2). Costs not available at 2011 price levels were inflated to 2011 using Dutch consumer price indices for all costs except for productivity costs per hour, which were inflated using Dutch collective labor agreement (CAO) price indices.

Costs for special education were calculated by determining the age-specific additional costs per child per year for special education compared to regular education [18], [22]. These costs were calculated for children younger than 16 years of age because in the Netherlands education is compulsory until that age. Indirect costs were calculated using the friction cost method [17] (appendix S2). A vaccine price of respectively €42.72 and €55.11 per dose for MenACWY and MenC were used according to the current pharmacy prices [23], [24].

### Vaccine Characteristics

Due to the lack of studies on the vaccine effectiveness (VE) of a MenACWY conjugate vaccine in the population and because the C component in MenACWY is immunologically non-inferior to that of MenC [25]–[28], the VE of MenACWY was assumed to be equal to the MenC vaccine. The VE was estimated by post-licensure studies about the MenC vaccine in the UK [29], [30]. VE was assumed to wane over time following equation 1:
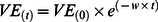
(1)


Where VE_(0)_ is the VE half a month after vaccination (percent), *w* is the annual waning rate and *t* is the time since protective immunity started (years). Full protection was assumed half a month after vaccination. The average duration of protection (1/*w*) was set at 4 years for vaccination at 14 months, based on the levels of protective antibody levels against serogroup C meningococcal disease among children 5 years after being vaccinated with the MenC vaccine at the age of 14 months [10]. For vaccination at 12 years, the average duration of protection was varied between 25 years (the base-case) and livelong protection. Experience with the MenC vaccine showed that for children vaccinated at the age of 12 years, protective antibody levels against serogroup C meningococcal disease were present in more than 80% of children 5 years after vaccination [10]. If the protective immunity would wane according to equation 1, approximately 80% of children would have protective antibody levels 5 years after vaccination, when the average duration of protection is set at 25 years.

### Herd Immunity

In the base-case analyses we conservatively assumed that the meningococcal C disease incidence would remain stable, despite waning of the vaccine efficacy, due to herd immunity and a low force of infection. For protection against serogroup A, W and Y it was assumed that vaccination with the MenC or MenACWY vaccine at 14 months would not induce herd immunity. Consequently, herd immunity effects in unvaccinated cohorts were assumed to be the same for MenC and MenACWY in the base-case, thereby applying herd immunity effects to the MenC incidence only.

For the strategy with MenACWY vaccinations at 14 months and 12 years, ICERs were calculated with and without herd-immunity.

Herd-immunity was incorporated in the model in a similar way as Rozenbaum *et al.* did [31].The magnitude of the herd-immunity was obtained by comparing the serogroup C incidence of 2001 with the average serogroup C incidence of 2007–2011. The incidence declined with 92% in both children between 0 and 1 years of age and in persons aged 27 years and over. As both groups were not protected by direct vaccination during both periods, this figure was used as the magnitude of herd-immunity induced by the vaccination. The decline in other age-groups, assuming a duration of protection of 5 years for vaccination at 14 months and of 25 years for vaccination at 12 years, was in accordance with a herd-immunity effect of 95% in age-categories that were also protected by direct vaccination and of 92% in all other age-groups.

For this scenario it was assumed that vaccination at 12 years would start at the same time as vaccination with 14 months. Therefore, a birth cohort, a 12-years old cohort and a cohort with the rest of the population was followed. Herd-immunity was accounted for only during the first year of vaccination, as it was assumed that to sustain the herd-immunity at the same level each year an additional shot at 12 years was needed, while we modelled only vaccinations in the first year of the model.

### Scenario Analysis

In scenario analysis we calculated what the extra value of MenACWY compared to MenC in euros was for vaccination at 14 months only at a threshold values of €20,000 and €50,000 per QALY.

Poor antibody persistence against meningococcal serogroup C disease following vaccination at a toddler age was observed in the UK [11]. Therefore, without additional vaccinations at a higher age, the herd immunity effect invoked by the catch-up campaign could disappear over time. Consequently, a possible restored circulation of the bacterium might occur in coming years [11]. Therefore, in a scenario analysis it was estimated what the cost-effectiveness was of MenACWY+MenACWY compared to vaccination with MenACWY at 14 months only. To simulate the effect of the additional vaccination at 12 years of age, it was assumed that the MenACWY+MenACWY strategy would result in maintenance of the current meningococcal C disease incidence. For the strategy with MenACWY at 14 months only it was assumed that this strategy would result in a serogroup C disease incidence similar to that seen during 2001, i.e. pre-MenC vaccination incidence levels minus the estimated direct effect of MenC vaccination at 14 months without a catch-up campaign. Consequently, for this scenario it was assumed that MenACWY has already been implemented and herd immunity against serogroup C disease was completely disappeared.

For the scenario analysis in which the disappearance of a herd-immunity effect would be prevented by vaccination at 12 years, direct and indirect vaccination effects were incorporated.

### Sensitivity Analysis

There are two key assumptions that determine the cost-effectiveness of MenACWY in comparison with MenC: the price difference and the vaccine effectiveness of both vaccines. Although the C component in the MenACWY has been shown to be immunologically non-inferior to that of MenC [25]–[28], the duration of protection after MenACWY vaccination is uncertain. In addition, although the list prices of both vaccines are known [23], [24], the reduced prices for the national immunisation programme might be different. Therefore, in bivariate sensitivity analysis, the influence of the price differential and the duration of protection on the ICER was assessed.

In univariate sensitivity analysis, relevant parameters were varied one at a time using the upper and lower limit of the 95% confidence intervals of the mean to determine which parameters had a great influence on the ICER. The vaccine price of MenACWY and the disease incidence were varied by 25%, as the degree of uncertainty for these parameters is unclear. Also, the vaccine effectiveness and discounting rates were varied one by one to explore their impact. Additionally, we varied the decay function for the vaccine effectiveness of the booster, thereby using the function previously used by De Wals *et al*. [32] and a linear function, thereby choosing the waning rate in accordance with the study of De Voer *et al.* [10].

Because the incidence of meningococcal disease is naturally fluctuating [33] a threshold analysis was performed to determine the overall incidence of serogroups A,C,W135,Y meningococcal disease which is required for vaccination to become cost-effective at €50,000 per QALY.

To assess the uncertainty of the ICERs, a probabilistic sensitivity analysis was performed. Parameters were generated using random sampling within the specified range of the corresponding distribution of the parameters. Outcome values were generated by running the model 10,000 times.

## Results

### Base-case

Vaccinating with MenACWY at 14 months would avoid 1 additional case of meningococcal serogroup A,W135,Y disease for the birth cohort followed for 99 years, corresponding to a gain of 2.9 QALYs (discounted), and was estimated to be cost-saving (table 3). Implementing an additional vaccination with MenACWY at 12 years of age would prevent 7 additional cases, which corresponds to a gain of 12 life years or 15 QALYs when compared with MenC at 14 months. This strategy has an additional total cost of about €9.5 million compared with MenC at 14 months and vaccination costs are about €20.6 million. The estimated ICER was €635,334 per QALY. Comparing this booster-dose strategy with MenACWY at 14 months, 6 additional cases of meningococcal disease are prevented, which corresponds to a gain of 9 life years and 12 QALYs (table 4). Additional total costs are approximately €11.7 million. The estimated ICER for this scenario was €988,490 per QALY.

**Table 3:** Incremental cost-effectiveness ratios and threshold analyses with and without herd-immunity against serogroup AWY.

	**ICER** a **[€/QALY (95% CI)]**		**Threshold incidence per 100,000 persons for €50,000/QALY**		
	**Base case incidence** a	**2011 incidence** b		**Base case** c	**2011 scenario** d
**No herd-immunity against AWY**					
MenACWY vs MenC	c.s. (c.s.-c.s.)	c.s. (c.s.-c.s.)		N.A.	N.A.
MenACWY+MenACWY vs MenC					
*Waning rate booster 0.04*	635,334 (259,672–1,121,856)	473,398 (179,847–849,293)		1.53	0.98
*No waning booster*	367,978 (151,968–649,963)	307,743 (114,982–554,719)		0.91	0.65
MenACWY+MenACWY vs MenACWY					
*Waning rate booster 0.04*	988,490 (440,833–1,675,575)	621,307 (251,868–1,089,972)		2.37	1.28
*No waning booster*	518,405 (224,848–887,791)	396,431 (160,174–700,855)		1.28	0.83
MenACWY+MenACWY vs MenACWY^e^					
*Waning rate booster 0.04*	c.s. (c.s. −3,168)	c.s. (c.s. −2,908)		N.A.	N.A.
*No waning booster*	c.s. (c.s. −3,269)	c.s. (c.s. −2,867)		N.A.	N.A.
**Herd-immunity against AWY**					
MenACWY+MenACWY vs MenC					
*Waning rate booster 0.04*	247,279 (101,873–426,876)	204,170 (76,617–365,845)		0.61	0.43
*No waning booster*	190,073 (75,665–331,131)	163,264 (59,144–293,917)		0.48	0.35
MenACWY+MenACWY vs MenACWY					
*Waning rate booster 0.04*	359,264 (166,041–593,227)	268,094 (110,297–466,305)		0.87	0.56
*No waning booster*	267,157 (119,263–447,724)	212,846 (84,860–372,550)		0.66	0.45

QALY = quality adjusted life year, c.s. = cost-saving, N.A. = not applicable.

a Base case incidence (average 2007–2011): 0.10 cases of serogroup A,W135,Y and 0.05 cases of serogroup C per 100,000 persons.

b 2011 incidence: 0.11 cases of serogroup A,W135,Y and 0.02 cases of serogroup C per 100,000 persons.

c Using the average age-distribution of the serogroup A,C,W135 and Y incidence of 2007–2011.

d Using the age-distribution of the serogroup A,C,W135 and Y incidence of 2011.

e Scenario analysis: compared with MenACWY at 14 months with only direct effects of vaccination and the incidence of C of 2001: 2.09 cases of serogroup C per 100,000 persons.

**Table 4:** Disease outcomes and costs associated with MenACWY vaccination at 14 months only and MenACWY vaccination at 14 months and 12 years.

	14 months	14 months +12 years		Δ
**Disease outcome**				
Meningococcal cases				
*All*	46		40	−6
*With skin scarring*	1		1	0
*With amputations*	0		0	0
*With neurological sequelae*	3		3	0
*Deaths*	3		3	0
Life years (disc.)	17,225,124		17,225,133	9
QALYs (disc.)	15,482,017		15,482,029	12
**Costs (€) (discounted)**				
Total costs^a^	9,356,182		21,039,738	11,683,555
Direct costs^b^	9,336,046		19,576,633	10,240,587
Vaccination costs^c^	8,729,539		19,123,583	10,394,044
Indirect vaccination costs^d^	1,959		1,450,110	1,448,151
Productivity losses	20,809		1,463,778	1,442,968

Δ = difference between the vaccination strategies in respectively disease outcome and costs,

disc. = discounted, QALY = quality adjusted life year.

a The total sum of all costs (including vaccination costs).

b Direct costs: total costs minus costs due to productivity losses.

c Vaccination costs: costs of the vaccine plus administration and overhead costs.

d Indirect vaccination costs: costs for the treatment of side effects of the vaccination. Productivity losses due to vaccination and side effects are included in ‘productivity losses’.

Assuming herd-immunity could also be invoked for serogroup AW135Y disease and lifelong protection after vaccination at 12 years or using the most recent disease incidence figures resulted in lower ICERs, but the ICERs for vaccination at 12 years of age were still too high to be considered cost-effective (table 3).

With the current low meningococcal C disease in the Netherlands, a schedule with vaccinations with MenC at 14 months and 12 years of age resulted in a high ICER of €2,620,329 per QALY, when compared to the current immunization schedule with a single vaccination with MenC at 14 months.

### Scenario Analysis

Since MenACWY at 14 months is cost-saving compared to MenC at 14 months mainly due to a lower vaccine price, we also estimated the extra value of MenACWY compared to MenC in euros for this scenario. MenACWY could cost €0.41 more than MenC for a threshold of €20,000 per QALY and €0.90 more at a threshold of €50,000 per QALY.

As the meningococcal serogroup Y disease incidence is currently increasing [19] we estimated for which disease incidence MenACWY+MenACWY would remain below €50,000 per QALY. Using the base-case assumptions and age-distribution, the total ACWY disease incidence should be 2.37 per 100,000 persons, when comparing MenACWY+MenACWY with MenACWY at 14 months. Using the age-distribution of 2011, this threshold incidence would be 1.28 per 100,000 persons (table 3). The scenario comparing MenACWY+MenACWY with MenACWY when herd immunity disappeared was estimated to be cost-saving (table 3).

### Bivariate and Univariate Sensitivity Analysis

In bivariate sensitivity analyses, when comparing MenC at 14 months with MenACWY at 14 months, the influence of a difference in the duration of protection of both vaccines and the price differential was assessed. Assuming that herd immunity is still present, MenACWY is still cost-saving when the average duration of protection for MenACWY is 1 year shorter than for MenC (3 vs. 4 year), when the vaccine price per dose is the same for both vaccines (table 5). However, if herd immunity is lost, and MenC has returned to pre-vaccination levels, vaccinating with MenACWY at 14 months only would result in a loss of QALYs if the duration of protection is 1 year shorter for MenACWY.

**Table 5:** Bivariate sensitivity analysis for MenACWY compared with MenC at 14 months.

	Incremental cost-effectiveness ratios [€/QALY (95% CI)]				
	Average duration of protection for MenACWY after vaccination at 14 months^a^				
*Price differential* b	ACWY: 6 y	ACWY: 5 y	ACWY: 4 y	ACWY: 3 y	ACWY: 2 y
- €5,-	c.s.	c.s.	c.s.	c.s.	€907,349^c^
- €1,-	c.s.	c.s.	c.s.	c.s.	€170,984^c^
€0,-	c.s.	c.s.	c.s.	c.s.	d
€1,-	€15,622	€25,162	€46,753	€143,724	d
€2,-	€44,576	€63,865	€107,335	€301,923	d
€3,-	€73,529	€102,569	€167,918	€460,122	d
€4,-	€102,483	€141,273	€228,501	€618,321	d
€5,-	€131,347	€179,976	€289,084	€776,520	d

QALY = quality adjusted life year, c.s. = cost-saving. Costve price differential indicates that MenACWY is cheaper than MenC. 14 months in a loss of QALYs if the duration of protect.

a The average duration of protection of MenC is held constant at 4 years.

b A negative price differential indicates that MenACWY is cheaper than MenC.

c ICER expresses the costs saved per QALY lossed.

d For these scenarios, MenACWY vaccination at 14 months costs more and saves less QALYs than MenC vaccination.

**Figure 2:**
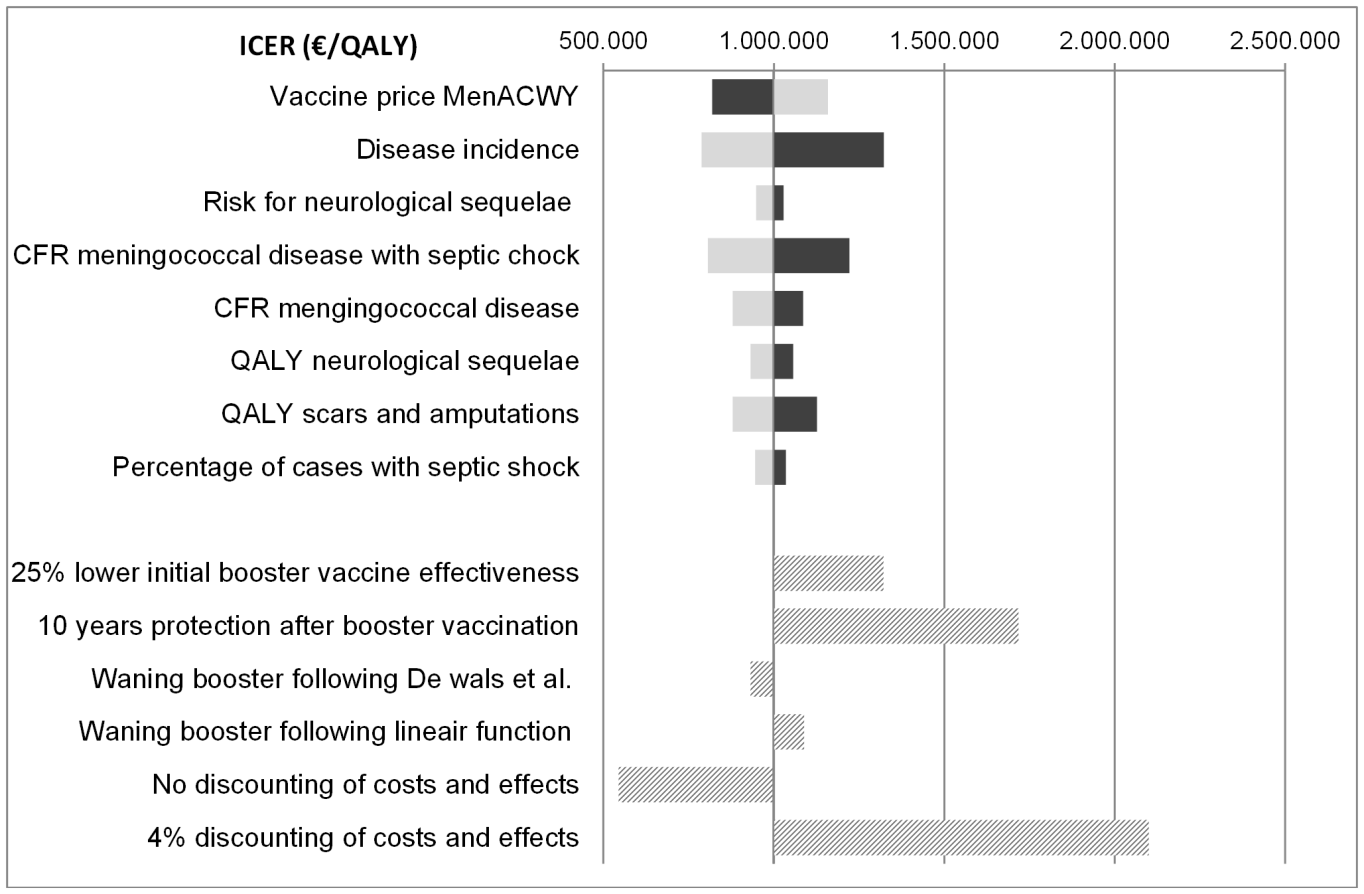
Univariate sensitivity analysis for MenACWY+MenACWY compared with MenACWY at 14 months. The above 7 parameters are varied 25% in both ways (dark bars: 25% decrease; light bars: 25% increase). ICER: incremental cost-effectiveness ratio, QALY: quality adjusted life year. CFR: case fatality rate.

Univariate sensitivity analyses, for the base-case MenACWY+MenACWY compared with MenACWY at 14 months, showed that the incidence of serogroup A,C,W135,Y disease had a high impact on the ICER. Also, the vaccine price of MenACWY and the case fatality rate had quite an impact on the ICER. No discounting of costs and effects substantially lowers the ICER, while applying a discount rate of 4% for both costs and effects dramatically increases the ICER (figure 2).

### Probabilistic Sensitivity Analysis

For the base-case, it is unlikely that vaccination with MenACWY+MenACWY could be considered cost-effective compared to MenC at 14 months as well as to MenACWY at 14 months. The scenario showing the prevention of a decline in herd immunity has a high probability to be considered cost-effective. Given the uncertainty of the model, 95% of simulations comparing MenACWY+MenACWY with MenACWY at 14 months were found below €1,750 per QALY and 100% below €15,800 per QALY.

## Discussion

Our analysis shows that vaccinating with MenACWY at 14 months is cost-saving compared with the current situation in the Netherlands. This is mainly due to the lower vaccine price of MenACWY compared with MenC. Also, the prevention of more cases of meningococcal disease with MenACWY contributes to the favourable ICER.

Using our base-case assumptions, adding a booster-dose at 12 years of age with MenACWY in addition to MenACWY at 14 months seems not to be a cost-effective strategy.

Serogroup Y meningococcal disease was rare in Europe but in recent years an increase in the incidence of serogroup Y meningococcal disease has been reported [34]. In the Netherlands, the incidence of serogroup Y meningococcal disease doubled during the last 5 years, but the number of cases is still low [15]. However, while during 2006–2010 on average 5% of all serogroup Y cases occurred in adolescents aged 12–19 years, in 2011 33% of serogroup Y cases occurred in this age-group with a likely high meningococcal transmission rate [15]. A further increase in the incidence of serogroup Y disease could lead to a more favourable ICER for the strategy with a booster-dose with MenACWY, as illustrated by our analysis with the epidemiologic data of 2011. By performing threshold analyses to determine for which meningococcal disease incidence the ICER remains below €50,000 per QALY, we enabled policy makers to make informed decisions about whether and when to implement a booster dose vaccination among adolescents.

In addition to a further increase in the incidence of serogroup Y disease, the potential loss of herd immunity against meningococcal C disease due to an unprotected adolescent population makes a booster dose at 12 years of age more favourable. Our scenario analysis shows that when herd immunity disappeared, MenACWY+MenACWY has the potential to be cost-effective. However, this scenario may be overrating since herd immunity will most likely disappear gradually. Moreover, part of the disease incidence reduction might be contributed to natural fluctuation. To estimate the effect of herd immunity and its disappearance more precisely, a transmission dynamic model should be used [35]. Force of infection is an important dynamic parameter for estimating herd immunity and in dynamic models the force of infection is allowed to change contrary to static models [36]. The scenario in which herd immunity is lost, does not seem unlikely because recent data from the UK suggests that protective antibody levels have declined markedly in all immunized cohorts since the time of vaccination [11]. Without high protective antibody levels a restored circulation of the bacterium is possible, although this has not been observed yet in the UK. However, it is difficult to predict when this might happen, just by the naturally fluctuating incidence of meningococcal disease [33].

For our scenario analysis, we assumed that vaccination at 14 months only would not be sufficient to maintain herd-immunity, because a dose under the age of 5 years is not likely to maintain herd immunity as little herd immunity is observed in Spain where the MenC catch-up program was till the age of 5 years [37]. In contrast, a booster-dose at 12 years is a good option for maintaining herd immunity, because vaccinating at around that age resulted in extended antibody persistence, providing protection in adolescents, among which meningococcal transmission rates is most likely high [9]. In addition, the response to a booster-dose with a MenC vaccine showed an age dependent trend in children at age of 6 to 12 years, with highest responses in 12 years old children [10], [11].

Experience with the MenC vaccine showed a reduction in serogroup C disease attack rates in unimmunized individuals and a significantly lower serogroup C carriage [6], [7]. Since it is uncertain whether a herd immunity effect for vaccination against serogroups A,W135,Y disease would be invoked by the implementation of vaccinations at 14 months and 12 years, we estimated ICERs with and without herd-immunity against serogroups A,W135,Y disease.

Data on the VE was estimated by post-vaccination studies which calculated VE by the screening method. Because this method uses data on the amount of cases in the population [38], a part of the herd immunity effect might be included in our VE estimate, possibly leading to an underestimation of the initial VE.

The clinical course and outcome of serogroup A, W-135 and Y disease was assumed to be equal to that of serogroup C disease, because limited data for serogroup A, W-135 and Y disease from the Netherlands is available. Two studies with a relatively low number of sero-group A, W-135 and Y cases indicate that serogroup W-135 and Y might be associated with a higher case-fatality rate [4], more sequelae, septicaemia, and in general a poorer outcome [39] than serogroup C or B. Therefore we may have underestimated the cost-effectiveness of the MenACWY vaccine.

Recently, it has been shown that three years after vaccination of toddles with MenACWY, antibody persistence was high for serogroups C, W-135 and Y, but was much lower for serogroup A [28]. Although this finding may have negative consequences on the ICER in countries with a high prevalence of serogroup A disease, it would not affect our results, since serogroup A disease has not been observed in the Netherlands during the past years [15].

Serogroup replacement by capsular switching due to vaccination was not modelled, because there is no evidence that vaccination with the MenC vaccine is an important driver in capsule switching of meningococci [40].

The MenACWY conjugate vaccine does not protect against serogroup B meningococcal (MenB) disease. Recently, a broad-coverage vaccine with the capacity to protect against MenB disease (4CMenB, Novartis) was licensed in the European Union [41]. Although more additional cases of meningococcal disease could be prevented in the Netherlands with the implementation of infant vaccination (2, 3, 4+11 mo schedule) against MenB disease (39 cases prevented in a single birth-cohort followed for 99 years in the base-case analysis) [42] than with the shift from a MenC vaccine to the MenACWY vaccine in the NIP, the latter option is more attractive from a cost-effective point of view [42], since the broad-coverage vaccine against MenB would be an additional vaccine instead of a replacement of the MenC vaccine. In addition, more vaccinations are needed with the MenB vaccine to confer protection in young children [28], [43] The recently licensed MenB vaccine has not only the capacity to protect against MenB disease; it might also protect against other meningococcal serogroups, because the protein antigens are not restricted to serogroup B meningococci [44]. The coverage against these other meningococcal serogroups is unclear and protection against these other serogroups is likely better with monovalent or quadrivalent conjugated vaccines specifically designed to protect against meningococcal serogroup A, C, W-135 and Y disease. Therefore, with the current knowledge, 4CMenB cannot replace routine infant vaccination with the MenC vaccine. Because the C component of MenACWY has been shown to be immunologically non-inferior to that of MenC [25]–[28], a shift from a MenC vaccine to this MenACWY vaccine in the NIP might be considered, especially when the meningococcal serogroup Y disease incidence keeps increasing in the Netherlands. To our knowledge, this is the first study which examines the cost-effectiveness of a MenACWY conjugate vaccine and of introducing a booster-dose with this vaccine at the age of 12 years in the Netherlands. Two cost-effectiveness studies on a MenACWY conjugate vaccine were performed in the United States [45], [46]. Both studies found that vaccinating children and adolescents would reduce substantially the burden of disease but at high costs. A third study is performed in Canada and is more similar to the Dutch situation because the comparable immunization program with respect to meningococcal disease [47]. This study showed an ICER of $31,000 per QALY for a booster-dose at 12 years with MenACWY in addition to MenC at 12 months, with MenC at 12 months as comparator. This ICER is much lower than the ICER calculated with this analysis in the Netherlands. This is mainly due to the higher incidence of serogroup W135 and Y disease in Canada [34], [47]. In any case, it is difficult to make a good comparison between these studies just by different discount rates (differential vs. equal discount rates for money and health) and differences in incidences in meningococcal disease.

### Conclusions

According to the results of this cost-effectiveness analysis, MenACWY at 14 months is cost-saving compared to MenC at 14 months, mainly due to a lower vaccine price. Adding a booster-dose with MenACWY at 12 years reduces the burden of disease but is not cost-effective with the current epidemiology. However, the scenario with the disappearance of herd immunity shows that the strategy with a booster-dose with MenACWY at 12 years has the potential to be cost-effective in the future. For a more precise estimation of the cost-effectiveness for the prevention of the loss of herd immunity, a dynamic model should be developed for the Netherlands.

## Supporting Information

Appendix S1 **Meningococcal serogroup ACWY disease incidences.**
(DOCX)

Appendix S2 **Indirect costs.**
(DOCX)

## References

[1] World Health Organization (2011) Meningococcal vaccines: WHO position paper, November 2011. Wkly Epidemiol Rec 86: 521–539. 22128384

[2] World Health Organization, SAGE Working Group (2011) Background paper on meningococcal vaccines. Available: http://www.who.int/immunization/sage/1_mening_background_document_v5_3__apr_2011.pdf. Accessed 22 June 2012.

[3] Oostenbrink R, Maas M, Moons KG, Moll HA (2002) Sequelae after bacterial meningitis in childhood. Scand J Infect Dis 34: 379–382. 12069024 10.1080/00365540110080179

[4] Scholten RJ, Bijlmer HA, Valkenburg HA, Dankert J (1994) Patient and strain characteristics in relation to the outcome of meningococcal disease: A multivariate analysis. Epidemiol Infect 112: 115–124. 8119350 10.1017/s0950268800057472 PMC2271487

[5] De Greeff SC, de Melker HE, Spanjaard L, Schouls LM, van Derende A (2006) Protection from routine vaccination at the age of 14 months with meningococcal serogroup C conjugate vaccine in the Netherlands. Pediatr Infect Dis J 25: 79–80. 16395110 10.1097/01.inf.0000195594.41449.c6

[6] Maiden MC, Ibarz-Pavon AB, Urwin R, Gray SJ, Andrews NJ, et al (2008) Impact of meningococcal serogroup C conjugate vaccines on carriage and herd immunity. J Infect Dis 197: 737–743. 18271745 10.1086/527401 PMC6767871

[7] Trotter CL, Maiden MC (2009) Meningococcal vaccines and herd immunity: Lessons learned from serogroup C conjugate vaccination programs. Expert Rev Vaccines 8: 851–861. 19538112 10.1586/erv.09.48 PMC3988355

[8] De Voer RM (2010) Meningococcal C specific immune responses: Immunity in an era of immunization with vaccine. Utrecht: Utrecht University.

[9] Christensen H, May M, Bowen L, Hickman M, Trotter CL (2010) Meningococcal carriage by age: A systematic review and meta-analysis. Lancet Infect Dis 10: 853–861. 21075057 10.1016/S1473-3099(10)70251-6

[10] De Voer RM, Mollema L, Schepp RM, de Greeff SC, van Gageldonk PG, et al (2010) Immunity against Neisseria meningitides serogroup C in the Dutch population before and after introduction of the meningococcal C conjugate vaccine. PloS One 5: e12144. 20730091 10.1371/journal.pone.0012144 PMC2921331

[11] Ishola DA Jr, Borrow R, Findlow H, Findlow J, Trotter C, et al (2012) Prevalence of serum bactericidal antibody to serogroup C Neisseria meningitides in England a decade after vaccine introduction. Clin Vaccine Immunol 19: 1126–1130. 22647271 10.1128/CVI.05655-11 PMC3416089

[12] Kaaijk P, van der Ende A, Berbers G, van den Dobbelsteen GP, Rots NY (2012) Is a single dose of meningococcal serogroup C conjugate vaccine sufficient for protection? Experience from the Netherlands. BMC Infect Dis 12: 35–2334–12–35. 22316426 10.1186/1471-2334-12-35 PMC3293716

[13] Ministry of Health Austria (2012) Immunization schedule Austria. Available: http://www.bmg.gv.at/cms/home/attachments/7/3/0/CH1081/CMS1286449898381/impfplan_2012_final,_1.2.2012.pdf. Accessed 2012 Jul 19.

[14] World Health Organization (2012) Immunization schedule Switzerland. Available: http://apps.who.int/immunization_monitoring/en/globalsummary/ScheduleResult.cfm. Accessed 2012 Jul 19.

[15] Netherlands Reference Laboratory for Bacterial Meningitis. Bacterial meningitis in the Netherlands. Annual reports 2007–2011. Amsterdam: University of Amsterdam, the Netherlands.

[16] European Medicines Agency (2012) EPAR-summary for the public: Nimenrex (EMA/CHMP/136315/2012). Available: http://www.ema.europa.eu/docs/en_GB/document_library/EPAR_-_Summary_for_the_public/human/002226/WC500127665.pdf. Accessed 2012 Dec 14.

[17] Hakkaart-van Roijen L, Tan SS, Bouwmans CAM (2010) Guidelines for costing research, methods and standardized prices for economic evaluations in health care. Diemen: Health Care Insurance Board.

[18] Statistics Netherlands. Available: http://statline.cbs.nl. Accessed 2012 Nov 15.

[19] European Union invasive bacterial infections surveillance network (2001) Invasive Neisseria meningitides in Europe –2001. Available: http://www.hpa-bioinformatics.org.uk/euibis/documents/2001_meningo.pdf. Accessed 2012 Dec 14.

[20] Welte R, van den Dobbelsteen G, Bos JM, de Melker H, van Alphen L, et al (2004) Economic evaluation of meningococcal serogroup C conjugate vaccination programmes in the Netherlands and its impact on decision-making. Vaccine 23: 470–479. 15530695 10.1016/j.vaccine.2004.06.019

[21] Stouthard MEA, Essink-Bot ML, Bonsel GJ, Barendregt JJ, Kramers PGN, et al.. (1997) Disability weights for disease in the Netherlands. Rotterdam: Department of Public Health, Erasmus University, the Netherlands.

[22] Dutch Ministry of Education Culture and Science (2012) Key figures 2007–2011. Available: http://www.government.nl/documents-and-publications/reports/2012/07/24/key-figures-2007-2011.html. Accessed 2012 May 16.

[23] College of Health Insurances. Pharmaceutical price meningococcal serogroup ACWY vaccine (Nimenrix®). Available: http://www.medicijnkosten.nl/. Accessed 2012 Jul 26.

[24] College of Health Insurances. Pharmaceutical price meningococcal serogroup C vaccine (Neisvac-C®). Available: http://www.medicijnkosten.nl/. Accessed 2012 Jul 26.

[25] Knuf M, Kieninger-Baum D, Habermehl P, Muttonen P, Maurer H, et al (2010) A dose-range study assessing immunogenicity and safety of one dose of a new candidate meningococcal serogroups A, C, W-135, Y tetanus toxoid conjugate (MenACWY-TT) vaccine administered in the second year of life and in young children. Vaccine 28: 744–753. 19887137 10.1016/j.vaccine.2009.10.064

[26] Knuf M, Pantazi-Chatzikonstantinou A, Pfletschinger U, Tichmann-Schumann I, Maurer H, et al. An investigational tetravalent meningococcal serogroups A, C, W-135 and Y-tetanus toxoid conjugate vaccine co-administered with Infanrix hexa is immunogenic, with an acceptable safety profile in 12–23-month-old children. Vaccine 29: 4264–4273. 21420417 10.1016/j.vaccine.2011.03.009

[27] Vesikari T, Karvonen A, Bianco V, van der Wielen M, Miller J (2011) Tetravalent meningococcal serogroups A, C, W-135 and Y conjugate vaccine is well tolerated and immunogenic when co-administered with measles-mumps-rubella-varicella vaccine during the second year of life: An open, randomized controlled trial. Vaccine 29: 4274–4284. 21443965 10.1016/j.vaccine.2011.03.043

[28] Vesikari T, Forsten A, Boutriau D, Bianco V, van der Wielen M, et al.. (2012) Randomized trial to assess the immunogenicity, safety and antibody persistence up to three years after a single dose of a tetravalent meningococcal serogroups A, C, W-135 and Y tetanus toxoid conjugate vaccine in toddlers. Hum Vaccin Immunother 8. doi: http://dx.doi.org/10.4161/hv.22166. 10.4161/hv.22166 PMC3656082 23032159

[29] Campbell H, Borrow R, Salisbury D, Miller E (2009) Meningococcal C conjugate vaccine: The experience in England and Wales. Vaccine 27 Suppl 2B20–9. 19477053 10.1016/j.vaccine.2009.04.067

[30] Campbell H, Andrews N, Borrow R, Trotter C, Miller E (2010) Updated postlicensure surveillance of the meningococcal C conjugate vaccine in England and Wales: Effectiveness, validation of serological correlates of protection, and modeling predictions of the duration of herd immunity. Clin Vaccine Immunol 17: 840–847. 20219881 10.1128/CVI.00529-09 PMC2863391

[31] Rozenbaum MH, Sanders EA, van Hoek AJ, Jansen AG, van der Ende A, et al (2010) Cost effectiveness of pneumococcal vaccination among Dutch infants: economic analysis of the seven valent pneumococcal conjugated vaccine and forecast for the 10 valent and 13 valent vaccines. BMJ 304: c2509. 10.1136/bmj.c2509 20519267

[32] De Wals P, Trottier P, Pépin J (2006) Relative efficacy of different immunization schedules for the prevention of serogroup C meningococcal disease: a model-based evaluation. Vaccine 24: 3500–3504. 16517032 10.1016/j.vaccine.2006.02.010

[33] Harrison LH, Trotter CL, Ramsay ME (2009) Global epidemiology of meningococcal disease. Vaccine 27 Suppl 2B51–63. 19477562 10.1016/j.vaccine.2009.04.063

[34] Halperin SA, Bettinger JA, Greenwood B, Harrison LH, Jelfs J, et al (2012) The chancing and dynamic epidemiology of meningococcal disease. Vaccine 30 Suppl2: B26–36. 10.1016/j.vaccine.2011.12.032 22178525

[35] Kauf TL (2010) Methodological concerns with economic evaluations of meningococcal vaccines. Pharmacoeconomics 28: 449–461. 20465314 10.2165/11535280-000000000-00000

[36] Drummond M, Chevat C, Lothgren M (2007) Do we fully understand the economic value of vaccines? Vaccine 25: 5945–5957. 17629362 10.1016/j.vaccine.2007.04.070

[37] Larrauri A, Cano R, Garcia M, Mateo S (2005) Impact and effectiveness of meningococcal C conjugate vaccine following its introduction in Spain. Vaccine 23: 4097–4100. 15908059 10.1016/j.vaccine.2005.03.045

[38] Chen RT, Orenstein WA (1996) Epidemiologic methods in immunization programs. Epidemiol Rev 18: 99–117. 9021306 10.1093/oxfordjournals.epirev.a017931

[39] Spanjaard L, Bol P, de Marie S, Zanen HC (1987) Association of meningococcal serogroups with the course of disease in the Netherlands, 1959–83. Bull World Health Organ 65: 861–868. 3124970 PMC2491086

[40] Trotter CL, Ramsay ME, Gray S, Fox A, Kaczmarski E (2006) No evidence for capsule replacement following mass immunisation with meningococcal serogroup C conjugate vaccines in England and Wales. Lancet Infect Dis 6: 616–617. 17008169 10.1016/S1473-3099(06)70584-9

[41] European Medicines Agency (2013) EPAR-summary for the public: Bexsero (EMA/755874/2012). Available: http://www.ema.europa.eu/ema/index.jsp?curl=pages/medicines/human/medicines/002333/human_med_001614.jsp&mid=WC0b01ac058001d124. Accessed 2013 Mar 5.

[42] Pouwels KB, Hak E, van der Ende A, Christensen H, van den Dobbelsteen GPJM, et al.. (2013) Cost-effectiveness of vaccination against meningococcal B among Dutch infants: crucial impact of changes in incidence. Hum Vaccin Immunother 9. doi: http://dx.doi.org/10.4161/hv.23888. 10.4161/hv.23888 PMC3899149 23406816

[43] Vesikari T, Esposito S, Prymula R, Ypma E, Kohl I, et al (2013) Immunogenicity and safety of an investigational multicomponent, recombinant, meningococcal serogroup B vaccine (4CMenB) administered concomitantly with routine infant and child vaccinations: results of two randomized trials. Lancet 381: 825–835. 23324563 10.1016/S0140-6736(12)61961-8

[44] Gill CJ (2013) Novel assessment of a novel meningitis B vaccine. Lancet Infect Dis. doi: http://dx.doi.org/10.1016/S1473-3099(13)70037-9. 10.1016/S1473-3099(13)70037-9 23414710

[45] Shepard CW, Ortega-Sanchez IR, Scott RD 2nd, Rosenstein NE, ABCs Team (2005) Cost-effectiveness of conjugate meningococcal vaccination strategies in the United States. Pediatrics 115: 1220–1232. 15867028 10.1542/peds.2004-2514

[46] Ortega-Sanchez IR, Meltzer MI, Shepard C, Zell E, Messonnier ML, et al (2008) Economics of an adolescent meningococcal conjugate vaccination catch-up campaign in the United States. CID 46: 1–13. 10.1086/524041 18171206

[47] De Wals P, Coudeville L, Trottier P, Chevat C, Erickson LJ, et al (2007) Vaccinating adolescents against meningococcal disease in Canada: A cost-effectiveness analysis. Vaccine 25: 5433–5440. 17560695 10.1016/j.vaccine.2007.04.071

[48] Medicines Control Agency (2000) Safety of meningococcal group C conjugate vaccines. Current Problems in Pharmacovigilance 26: 14.

[49] Van Lier EA, Oomen PJ, Giesbers H, Drijfhout IH, de Hoogh PAAM, et al.. (2012) Immunization coverage National Immunization Programme in the Netherlands: Year of report 2012. Report no. 201001001, National Institute of Health and the Environment, Bilthoven, the Netherlands.

[50] Neppelenbroek SE, de Vries M, Greeff SC, Timen A (2002) ‘da’s goed gedaan? Woordverslag van de landelijke vaccinatiecampagne meningokokken C. ISBN 90–72779–38-X. 2003. GGD Nederland.

[51] De Greeff SC, de Melker HE, Schouls LM, Spanjaard L, van Deuren M (2008) Pre-admission clinical course of meningococcal disease and opportunities for the earlier start of appropriate intervention: a prospective epidemiological study on 752 patients in the Netherlands, 2003–2005. Eur J Clin Microbriol Infect Dis 27: 985–992. 10.1007/s10096-008-0535-1 18493804

[52] Van Deuren M, Brandtzaeg P, van der Meer JW (2000) Update on meningococcal disease with emphasis on pathogenesis and clinical management. Clin Microbiol Rev 13: 144–166. 10627495 10.1128/cmr.13.1.144-166.2000 PMC88937

[53] Dutch Healthcare Authority. Medical specialized treatment and its tariffs 2011. Available: http://www.nza.nl/137706/145406/BR-CU-2015_bijlage_2_med.spec.behandelingen-en-tarieven_2011.xls. Accessed 2012 May 28.

[54] Dutch Association of Hospitals. Number of general hospitals to bed numbers. Available: http://www.nvz-ziekenhuizen.nl/feiten_en_cijfers/FAQ#beddenaantal. Accessed 2012 May 28.

[55] Kind P, Hardman G, Macran S. (1999) UK population norms for EQ-5D. Discussion paper 172. The University of York Centre for Health Economics. Available: http://www.york.ac.uk/inst/che/pdf/DP172.pdf. Accessed 2012 Jun 1.

