# Supplementary material for: Meningococcal Serogroup A, C, W135 and Y Conjugated Vaccine: A Cost-Effectiveness Analysis in the Netherlands
Source: PLoS One. 2013 May 31;8(5):e65036. doi: 10.1371/journal.pone.0065036 (PMC3669019; doi:10.1371/journal.pone.0065036)
Supplement: Appendix S1 — Meningococcal serogroup ACWY disease incidences. (DOCX) [file pone.0065036.s001.docx]

**Appendix S1. Meningococcal serogroup A,C,W_135_ and Y disease incidences**

This appendix presents the age group specific incidence of meningococcal serogroups A,C,W135 and Y disease in the Netherlands [1].

Figure S1 shows the average incidences of serogroups C,W135,Y meningococcal disease of 2007-2011 and 2011 separately. Serogroup A disease has not been observed in the corresponding period. For the base-case, the average incidence over 2007-2011 for C,W135,Y was used. For scenario analyses, we used the most recent incidence (2011), as serogroup Y meningococcal disease is currently increasing, especially in adolescents.

Figure S1 shows that while using the average incidence of 2007-2011 no clear peak was observed in adolescents, using only data from 2011 an increase in the incidence of serogroups W and Y was observed among 15-19 years old. The majority of these cases were caused by serogroup Y disease (83%).

The higher disease incidence in serogroup W and Y disease in 2011 is almost solely explained by an increase in serogroup Y disease, as the incidence of serogroup W disease was lower in 2011 than in the years before (1 reported case in 2011 vs. on average 5 cases per year in 2006-2010).

The serogroup Y disease incidence is steadily increasing over the recent years, from 4 reported cases in 2006 to 15 reported cases in 2011.

Figure S2 shows that the incidence of serogroup C disease in 2001 (note the different y-axis) was highest among young children till the age of 19 years, with peaks observed in the age groups under the age of 1 years, 1 to 4 years and 15 to 19 years. This figure further shows that the incidence of serogroup C meningococcal disease has decreased substantially after 2001 and is approximately 10 years after the catch-up campaign at a low level.

**References**

1. Netherlands Reference Laboratory for Bacterial Meningitis. Bacterial meningitis in the Netherlands. Annual reports 2006-2011. Amsterdam: University of Amsterdam, the Netherlands.

**Figure S1.** **Meningococcal disease incidence per 100,000 persons per age group**. The average incidences of serogroup C and serogroup W135, Y during 2007-2011 are shown. The average incidence in 2011 is also shown separately. Incidences are corrected for underreporting.

**Figure S2. Serogroup C meningococcal disease incidence per 100,000 persons per age group.** The average incidences of serogroup C during 2001, 2007-2011 and 2011 are shown. Incidences are corrected for underreporting.
