# Supplementary material for: Meningococcal Serogroup A, C, W135 and Y Conjugated Vaccine: A Cost-Effectiveness Analysis in the Netherlands
Source: PLoS One. 2013 May 31;8(5):e65036. doi: 10.1371/journal.pone.0065036 (PMC3669019; doi:10.1371/journal.pone.0065036)
Supplement: Appendix S2 — Indirect costs. (DOCX) [file pone.0065036.s002.docx]

**Appendix S2. Indirect costs**

This appendix describes the method which is used for estimating indirect costs, which is the friction cost method. The friction time is the estimated vacancy period plus four weeks. This resulted in a friction time of 115 days [1]. This friction time was taken into account for meningococcal cases which resulted in death and for 50% of the cases with neurological sequelae [2]. Parents lose work hours when accompanying children to vaccination sites or health care providers. In the Netherlands, both parents work in 68.6% of families and the employed workforce consists of 41.2% part-time workers [3]. The chance that a full-time working parent takes off to bring a child for vaccination or to the GP is therefore 40.3%. For bringing a child to vaccination for a booster-dose, 1 hour productivity loss was taken into account. For an adverse event the productivity loss was 1.5 hour and for an anaphylactic reaction, 1 day of productivity loss was taken into account [2].

For cases under the age of 15 years, a work-loss of 3 days for parents was taken into account for both meningococcal disease with or without septic shock. For cases 15 years or older a work-loss of 16 days was taken into account for meningococcal disease without septic shock and 19 days for cases with a septic shock. The outcomes scars and amputations were associated with an additional work-loss of respectively 2 and 8 days for cases 15 years and older [2].

Productivity costs per hour were derived from the Dutch guidelines for pharmacoeconomic research [4]. For cases older than 15 years, productivity costs per hour were used from the corresponding age category total men and women. For productivity costs due to productivity loss by parents, the average age- and sex-specific production value per person was weighted with the respective age-specific probability of becoming a mother or a father [3].

**References**

1. UWV Work Company. (2010) Vacancies in the Netherlands 2010. The vacancy market and recruitment into view. Available: <http://www.uwv.nl/overuwv/Images/Vacatures%20in%20Nederland%20in%202010.pdf>. Accessed 28 May 2012.

2. Welte R, van den Dobbelsteen G, Bos JM, de Melker H, van Alphen L, et al. (2004) Economic evaluation of meningococcal serogroup C conjugate vaccination programmes in the Netherlands and its impact on decision-making. Vaccine 23: 470-479.

3. Statistics Netherlands. Available: <http://statline.cbs.nl>. Accessed 15 November 2012.

4. Hakkaart-van Roijen L, Tan SS, Bouwmans CAM. (2010) Guidelines for costing research, methods and standardized prices for economic evaluations in health care. Diemen: Health Care Insurance Board.
